# NEIGHBORHOOD SOCIAL AND SERVICE ORGANIZATIONS AND COGNITIVE DECLINE FOR OLDER MEXICAN AMERICANS

**DOI:** 10.1093/geroni/igae098.0485

**Published:** 2024-12-31

**Authors:** Ji Hyun Lee, Sunshine Rote, Elham Mahmoudi, Marina Armendariz, Toni Antonucci, Mark Manning

**Affiliations:** Montana State University, Bozeman, Montana, United States; University of Louisville, Louisville, Kentucky, United States; University of Michigan Medical School, Ann Arbor, Michigan, United States; The University of Texas San Antonio, San Antonio, Texas, United States; University of Michigan, Ann Arbor, Michigan, United States; Oakland University, Rochester, Michigan, United States

## Abstract

Neighborhood resources are salient in healthy brain aging. Neighborhood “third places” such as community centers and local eateries are increasingly recognized for their role in supporting the cognitive health of older residents. However, less is known about whether cognitive benefits from neighborhood resources are experienced similarly across diverse older adults. In the current study, we examined the associations between the availability of neighborhood social (i.e., religious institutions, social clubs) and service organizations (i.e., senior centers, community food banks) on cognitive decline among Mexican Americans living in urban and rural areas in the southwest U.S. region. We linked the panel data from the 2004-2013 Hispanic Established Populations for the Epidemiologic Study of the Elderly (H-EPESE) with the census-level data on the number of organizations from National Neighborhood Data Archive (N= 628, Age M(SD) = 80 (3.6), female 64%, U.S. born 54%). Cognitive decline was based on MMSE scores with cut-off points adjusted for low educational attainment. The generalized estimating equations model accounted for person-level covariates such as age, gender, years of education, marital status, nativity, and dual eligibility for Medicaid and Medicare. We found that, in urban settings, the presence of religious organizations was associated with better cognition, but a higher number of civic organizations were linked to worse cognitive health and a steep decline in cognition. Social and service organizations were not associated with cognition in rural settings. Our findings highlighted the importance of urban/rural contexts in identifying neighborhood organizations that may support healthy brain aging for older Mexican Americans.

